# Mechanisms of the Rapid Effects of Ketamine on Depression and Sleep Disturbances: A Narrative Review

**DOI:** 10.3389/fphar.2021.782457

**Published:** 2021-12-14

**Authors:** Bijia Song, Jun-Chao Zhu

**Affiliations:** Department of Anesthesiology, Shengjing Hospital of China Medical University, Shenyang, China

**Keywords:** sleep disturbances, ketamine, antidepressant, depression, neurocognition

## Abstract

Recently, sleep has been recognized as a crucial factor for health and longevity. The daily sleep/wake cycle provides the basis of biorhythm, which controls whole-body homeostasis and homeodynamics. Sleep disturbances can contribute to several physical and psychological disorders, including cardiovascular disease, obesity, depression, and cognitive dysfunction. The clinical use of the N-methyl-D-aspartate (NMDA) receptor antagonist ketamine began in the 1970s. Over the years, physicians have used it as a short-acting anesthetic, analgesic, and antidepressant; however, in-depth research has revealed new possible applications for ketamine, such as for treating sleep disturbances and circadian rhythm disorders. The aim of this narrative review is to examine the literature on the mechanistic role of the antidepressant ketamine in affecting sleep disturbance. Additionally, we discuss the pharmacologic and pharmacokinetic mechanisms of ketamine as an antidepressant and the predictive biomarkers for ketamine’s effect on sleep and cognitive function.

## Introduction

Sleep is a dynamic state characterized by behavioral, physiological, and electrophysiological changes. Behavioral changes include an increase in the sensory threshold of external stimuli, stillness, species-specific sleep posture, and reversibility. According to the electrophysiological activities of the brain and surrounding muscles, sleep is classified into two phases that alternate during the night cycle: rapid eye movement sleep (REM) and non-rapid eye movement sleep (NREM) (McCarley, 2011; Scammell et al., 2017; Eban-Rothschild et al., 2018). Some changes occur in the sleep pattern with increase in age, including reduced total sleep time and efficiency, increased sleep fragmentation, greater difficulty falling asleep, short eyesight exercise (REM) sleep and slow wave sleep (Wolkove et al., 2007; Pace-Schott and Spencer, 2011). Insufficient sleep and sleep disorders are associated with adverse health outcomes, including obesity, diabetes, chronic pain or pain sensitivity, cardiovascular disease, cancer, stress, neurocognitive dysfunctions, and psychiatric symptoms (Yaremchuk, 2018; Mehra, 2019). Moreover, basic neuroscience research has shown that prolonged chronic stress and depression-like behaviors are associated with impaired neuroplasticity. Sleep disturbances as one of the prominent symptoms of impaired neuroplasticity and are highly prevalent in mental disorders, such as major depressive disorder (MDD), bipolar disorder, post-traumatic stress disorder, and generalized anxiety disorder; additionally, sleep disturbances are associated with poor cognitive, emotional, and interpersonal function (Kahn et al., 2013; Rasch and Born, 2013). Sleep deprivation caused by psychological problems may lead to changes in the secretion of neuroinflammatory factors and neurotransmitter activity (such as adenosine) and brain hypoxia or hypoperfusion injury, and consequently potentiate neuronal apoptosis in cognition-related brain regions (Gulia et al., 2018). Ketamine, an N-methyl-D-aspartate (NMDA) antagonist has been reported to exert an antidepressant effect, which occurs as early as 40 min after administration and usually lasts for 7 days in patients with treatment-resistant major depression (TRD), therefore representing a new therapeutic option for treating depression (Moaddel et al., 2013; Kryst et al., 2020). Although studies have shown that ketamine exerts neuroprotective activity, studies on its rapid antidepressant effects on sleep disturbances and circadian dysregulation are limited. Treatments for sleep disorders may improve sleep-related diseases by aligning physical and psychological functions with circadian sleep/wake rhythm. Therefore, in this narrative review, we examine the literature on the internal relationship between sleep, cognitive function and depression (Table 1), the consequences of sleep disturbances (Table 2) and the effect of ketamine on sleep disturbances (Table 3). Additionally, pharmacology and pharmacokinetics of ketamine and predictive biomarkers of ketamine’s effect on sleep and cognitive function are also discussed (Table 3).

**TABLE 1:** The internal relationship between sleep, cognitive function and depression.

The internal relationship between sleep, cognitive function and depression	Mechanisms	References
Sleep disturbances in depression	Sleep deficiency increases the transcription of IL-6 and TNF by activating nuclear factor-kappaB (NF-κB) and then contributes to increased levels of inflammatory cytokines (eg IL-6 and TNF) throughout the day, which could finally lead to depression	Irwin et al. (2006)
		Irwin et al. (2008)
		Slavich and Irwin (2014)
	Patients diagnosed with major depressive disorder (MDD) presented abnormal genetic regulation of serotonergic transmission and levels of serotonin metabolites and NE have been shown to be decreased, which then caused the disruption of REM sleep of patients with MDD.	Krishnan and Nestler (2008)
		Caspi et al. (2010)
		Wang et al. (2015)
	Glutamate signaling also plays an important role in sleep, in particular, during the thalamocortical slow oscillations of non-REM (NREM) sleep. Glutamate deficiency could lead to sleep disturbances and low levels of glutamate lead to cell death in areas of the brain responsible for mood regulation	Peterson and Benca (2008)
	The dysregulation of clock genes caused by sleep disturbance and environmental factors cause the abnormal expression of clock genes was considered as an important factor associated with the development of both insomnia and depression	Monteleone et al. (2011)
Cognitive dysfunction in MDD	Information processing of patients with MDD requires a stronger and longer period of activity of the amygdala. Increased activity of the amygdala frequently occurs in combination with an increased concentration of noradrenaline and cortisol that are responsible for memory enhancement, which could further explain the tendency of MDD patients towards a continuous and excessive focusing on negative memories	Stuhrmann et al. (2013)
	The most crucial area of the cerebral cortex involved in the production of cognitive deficits is the anterior cingulate cortex (ACC), which includes ventral and dorsal anterior cingulate cortex (vACC and dACC) and integrates neuronal circuits responsible for emotion processing and affect regulation. In the course of MDD cellular abnormalities in the cerebral and sub-cerebral structures disrupt monoaminergic transmission which showed a reduced activity of the vACC and resulted in a weaker stimulation of dopamine secretion in the limbic system which is the major neurotransmitters in MDD.	Drevets et al. (2008)
—	Through responsing to chronic stress, the development of MDD has been facilitated and then the stimulation of the immune system is enhanced, which leads to an increase in the level of inflammatory mediators, such as IL-1β, IL-6, BDNF and TNF-α (tumor necrosis factor), and results in the activation of microglia. Then the persistence of an inflammatory response leads to impaired cognitive function	Rogers et al. (2011)

**TABLE 2:** The consequences of sleep disturbances.

Consequences of sleep disturbances	—	—
Neurodegenerative disease	Sleep disturbances were suggested to inhibits the inflow of apolipoprotein E (APOE) in cerebrospinal fluid (CSF) and clearance of APOE in interstitial fluid (ISF), which reduces the removal of Aβ in CSF and increase cerebral Aβ deposition. Aβ plaques also trigger the mislocalization of aquaporin 4 (AQP4) and decrease CSF influx, thus forming a vicious circle. And apnoea or obstructive sleep apnea syndrome was related to higher levels of AD-related neuronal injury biomarkers (ie, P-Tau and T-Tau)	Branger et al. (2015)
		Lucey et al. (2017)
		Carvalho et al. (2018)
		Lucey et al. (2018)
		Peng et al. (2016)
		Hooghiemstra et al. (2016)
		Diaz et al. (2017)
	Furthermore, PD dementia is caused by the aggregation of the protein α-synuclein, deposits of which are known as Lewy bodies (DLBs). Sleep disturbances could raise the level of DLBs that aggravate the development of PD.	Barone et al. (2009)
	Sleep apnea-associated intermittent brain hypo-oxygenation and inflammation may accelerate the degenerative process in already vulnerable or affected nigral dopaminergic neurons. As such, sleep apnea may exacerbate or accelerate the clinical manifestation of prodromal PD.	Bohnen et al. (2019)
Cardiovascular disease	Inflammation is associated with the occurrence and development of cardiovascular diseases. Experimental sleep restriction is associated with acute increases in the activity of upstream pro-inflammatory molecular pathways [e.g., Tumor Necrosis Factor-α (TNFα) messenger RNA and nuclear factor (NF)-κβ activation]	Irwin et al. (2011)
		Willeit et al. (2016)
	Short sleep duration increases cardiovascular disease risk though autonomic dysfunction, experimental sleep deprivation has been shown to decrease parasympathetic activity and increase sympathetic activity, indexed by high-frequency heart rate variability and plasma norepinephrine, respectively	Tobaldini et al. (2013)
	Chronic metabolic dysfunction in the form of insulin resistance and impaired glucose tolerance is a leading risk factor for cardiovascular disease morbidity and mortality. Subsequent experimental and observational studies suggested that sleep curtailment is a critical risk factor for the development of obesity, diabetes	Huang et al. (2014)
		Killick et al. (2015)
Cognitive function	The link between sleep disturbances and cognitive decline and dementia may be related to cortical thinning, a marker of cortical atrophy found in many dementia subtypes, that decreased cortical thickness in the lateral orbitofrontal cortex and inferior frontal gyrus was associated with increased sleep fragmentation as measured by an Actigraph, and the atrophy of grey matter in the medial prefrontal cortex (mPFC) is associated with attenuated SOs-spindles coupling and impairment of hippocampal-dependent memory in the elderly	Zimmerman and Aloia (2006)
		Macey et al. (2008)
		Mander et al. (2013)
		Zarei et al. (2013)
		Möller et al. (2016)
	Shorter sleep duration may also contribute to cognitive decline through degeneration of the hippocampus through multiple pathways, including changes in neuronal excitability, decreasing synaptic plasticity, and decreasing neurogenesis. Sleep deprivation activates neurotoxic complement components C3a and C5a, which disturb the hippocampal brain-derived neurotrophic factor (BDNF) pathway and adult neurogenesis, eventually impairing spatial memory	Kessler et al. (2019)
	Acute sleep deprivation reduces dendritic spine density in the hippocampal neurons and results in long-term memory impairment, while recovery sleep ameliorates spine loss and memory impairment	Havekes et al. (2016)

**TABLE 3:** Potential mechanisms of ketamine in improving sleep quality.

Potential mechanisms of ketamine in improving sleep quality	Mechanisms	References
Antidepressant	(R,S)-ketamine can selectively block NMDA receptor expressed on GABA inhibitory interneurons, resulting in a decreased activity of GABAergic interneurons, and de-inhibition of pyramidal neurons, which further increases excitatory neurotransmitter glutamate released from the synaptic cleft, activates the AMPAR, and increases the level of BDNF which finally increase early sleep slow-wave activity during non-REM sleep	Bjorkholm and Monteggia (2016)
		Aleksandrova et al. (2017)
		Duncan et al. (2017)
	The level of brain-derived neurotrophic factor depends on the regulation of eukaryotic elongation factor 2. Phosphorylated eukaryotic elongation factor 2 can inhibit the translation of brain-derived neurotrophic factor. Ketamine promotes the translation of brain-derived neurotrophic factor by reducing eukaryotic elongation factor 2 kinase activity and inhibiting the phosphorylation of eukaryotic elongation factor 2	Bjorkholm and Monteggia (2016)
		Aleksandrova et al. (2017)
		Duncan et al. (2017)
	(R,S)-ketamine may significantly increase the release of monoamine transmitters in the central nervous system, promote angiogenesis and synaptic regeneration, and enhance neuronal activity, which may be associated with its antidepressant effects	Ago et al. (2019)
Regulate sleep and circadian system	Ketamine has well-described rapid antidepressant effects in clinical studies of individuals with treatment-resistant MDD, one of the possible antidepressant mechanisms of ketamine may be associated with its actions on clock-gene-related molecules, leading to alterations in the circadian timekeeping of the central clock, and/or with its efects on entrainment circuits that synchronise the central clock with external lighting cycles	Krenk et al. (2012)
		Cheng et al. (2018)
		Duncan et al. (2017)
	Ketamine could alter the timing and amplitude of circadian rhythms in rapid responders with increased total sleep, REM sleep, SWA, and slow-wave sleep, potentially. Ketamine’s effects on sleep EEG were specific to low frequencies corresponding to the SWA range. Further, ketamine did not increase SWA in waking epochs prior to sleep onset, indicating that the change was sleep-specific. Negligible effects of the ketamine infusion on sleep EEG were measured in bands corresponding to sleep spindles, and alpha or theta frequencies	Duncan et al. (2019)
		Duncan et al. (2013)
	Ketamine-induced changes in BDNF levels are associated with its antidepressant efects and increased early sleep SWA during non-REM, as well as with the improvement in sleep quality in subjects with treatment-resistant depression	Homberg et al. (2014)
		Duman et al. (2014)
		Duncan et al. (2017)
Neurocognitive effect	Ketamine have significant effect on the cerebral activity of in animals and humans. The cognitive effect of the ketamine may be related with the ability to inhibit the cerebral metabolic rate of the oxygen which causes reduction of excitatory amino acid glutamate neurotransmitter release. Therefore, administration of ketamine and other N-methyl-D-aspartate receptor antagonists, such as memantine, is used to improve the symptoms AD.	Masliah et al. (1996)
		Mallory et al. 1997
	Oxidative stress and protein damage are the processes related to the pathogenesis of AD. The pharmacological effect of ketamine preventsprotein denaturation, lipid peroxidation and secondary damage of neuronal cells via reducing the formation of free radicals and release of various inflammatory mediators, which may be the other reason to improve AD.	Lewis et al. (2012)
		Ye et al. (2013)
	Ketamine presented the significant lower apoptosis rate by the study of the hippocampal neuronal apoptosis, which is accordance with the change in the memory ability and spatial learning in rats. These results have confirmed that the ketamine can inhibit the anesthesia on the rat cognitive lies of the hippocampal neuronal apoptosis	Sun et al. (2011)
	Ketamine was found to induce neurodegeneration in the developing brain, which led to heated discussions on the neurotoxicity of ketamine use in children. Further studies have indicated that high doses or repeated ketamine doses can induce cell death, especially apoptosis, in many kinds of *in vivo* and *in vitro* models from mice, rats, and monkeys. Ketamine was also found to disturb normal neurogenesis of neural stem progenitor cells (NSPCs) in the developing brain	Dong and Anand. (2013)
		Ikonomidou et al. (1999)
		Fredriksson et al. (2004)
		Amr (2010)
		Dong et al. (2012)

## The Relationship Between Sleep, Cognitive Function, and Depression

### Sleep Disturbances in Depression

A large and occasionally contradictory body of literature has described the association of sleep disorders with MDD. Polysomnographic studies have shown that MDD is associated with several sleep problems, including insomnia (88%), hypersomnia (27%), sleep disordered breathing, restless legs syndrome (RLS), and abnormal sleep architecture, such as prolonged sleep onset latency, frequent nocturnal awakenings, and poor sleep efficiency. Decreased REM sleep latency and prolonged REM sleep periods early in the night occur in depressed patients with sleep disturbance, leading to an overall increase in the proportion of REM sleep (Benca et al., 1992; Yates et al., 2004). The relative excess of REM sleep seems to come at the expense of stage N3 sleep, also known as slow wave sleep. Apart from less time spent in slow wave sleep in depressed patients compared with controls, there is an abnormal distribution of slow wave activity (SWA), a marker of SWS intensity, in patients with depression (Kupfer et al., 1990). Moreover, the relationship between sleep problems, and mood symptoms is bidirectional, in that poor sleep can precede an episode of major depressive disorder, and depressed mood can disrupt normal sleep pattern (Fang et al., 2019).

Sleep deficiency increases the transcription levels of IL-6 and TNF by activating nuclear factor-kappaB (NF-κB), resulting in increased levels of inflammatory cytokines (e.g., IL-6 and TNF) throughout the day (Irwin et al., 2006; Irwin et al., 2008). Moreover, studies have shown a strong relationship between inflammation and depression, as evidenced by the higher levels of inflammatory markers in depressed patients compared with non-depressed individuals, and patients with inflammatory disorder also showed a high rate of comorbid depression (Slavich and Irwin, 2014). Furthermore, the mutual effect of cholinergic and monoaminergic neurons regulates the onset of REM sleep with a rapid decrease in monoamines, such as serotonin (5-HT), norepinephrine (NE) and dopamine, and a concomitant increase in cholinergic tone. Abnormal genetic regulation of serotonergic transmission, abnormal levels of serotonin metabolites, and decrease in NE level result in fragmented REM sleep in patients with MDD (Krishnan and Nestler, 2008; Caspi et al., 2010; Wang et al., 2015). Additionally, glutamate signaling plays an important role in sleep, particularly during the thalamocortical slow oscillations of non-REM (NREM) sleep. Glutamate deficiency could lead to sleep disturbances and cell death in areas of the brain responsible for mood regulation (Peterson and Benca, 2008). Circadian rhythm is a 24-h rhythm in physiology and behavior controlled by molecular clocks in suprachiasmatic nuclei (SCN), and it plays an important role in sleep duration, continuity, and architecture. Abnormal expression of clock genes resulting from sleep disturbance and environmental factors is associated with the development of both insomnia and depression (Monteleone et al., 2011).

### The Association of Cognitive Dysfunction With MDD and the Underlying Pathological Mechanism

MDD is the leading cause of disability worldwide with a high public health burden. At the molecular level, depression involves neuroplasticity failure, including neuronal atrophy and synaptic depression in the medial pre-frontal cortex (mPFC) and hippocampus (Abdullah et al., 2015; Duman et al., 2016). At the neurocognitive level, depression is defined as impaired cognitive flexibility and prefrontal inhibition, which could lead to inflexible negative biases in cognition, such as rigidly held negative beliefs (Disner et al., 2011). A comparative study involving 22 nonpsychotic MDD patients and 30 healthy participants showed significantly diminished attention span, working memory, verbal long-term memory, and verbal fluency in MDD patients compared with normal patients (Landro et al., 2001). Similarly, depressed patients scored more than 1.5 standard deviations lower in attention and working memory, 1.0 standard deviation lower in verbal long-term memory, and 0.5 standard deviation lower in verbal fluency (Beblo et al., 1999; Leuchter et al., 2004). Moreover, the relationship between MDD and global cognitive function may be modulated by the severity of depression. Rohling et al. (2002) reported greater cognitive difficulties in both automatic and effortful attention in patients hospitalized for depression. Additionally, depression may negatively impact different types of memory, including explicit, implicit, short term, long term, and working memory (Wang et al., 2006). Therefore, research into their pathological mechanisms may facilitate better insight into the neurocognitive dysfunction of depression. MDD patients with low information processing by the amygdala usually exhibit cognitive distortions (increased responsivity to negative information) (Stuhrmann et al., 2013). Increased activity of the amygdala frequently occurs in combination with increased concentration of noradrenaline and cortisol, which are responsible for memory enhancement. Additionally, the most crucial area of the cerebral cortex involved in the production of cognitive deficits is the anterior cingulate cortex (ACC), which includes ventral and dorsal anterior cingulate cortex (vACC and dACC). The ACC integrates neuronal circuits responsible for emotion processing and affects regulation. During MDD, there is a decrease in the activity of the vACC, resulting in a weaker stimulation of dopamine secretion in the limbic system, which is the major neurotransmitters in MDD; moreover, the decrease in vACC activity is due to cellular abnormalities in the cerebral and sub-cerebral structures, which disrupt monoaminergic transmission (Drevets et al., 2008). Chronic stress can facilitate the development of MDD by inducing the level of inflammatory mediators, such as IL-1β, IL-6, BDNF, and TNF-α (tumor necrosis factor), which can result in the activation of microglia (Réus et al., 2018). Moreover, microglia activation is a response for decrease in the level of CX3CL1 protein, a chemokine that regulates neuroimmunization, and participates in synaptic plasticity and cognitive function regulation. Additionally, increased expression of IL-1β is related to increased rate of nerve cell mortality, predominantly in the hippocampus, which has the highest IL-1β binding sites (Rogers et al., 2011).

## Consequences of Sleep Disturbances

### Effect and Mechanism of Sleep Disturbances on Neurodegenerative Disease

Neurodegenerative diseases are characterized by progressive deterioration of brain structure and function. Additionally, degeneration of selective neuron populations can lead to cognitive disorders [as observed in Alzheimer’s disease (AD) and frontotemporal dementia] or major motor incoordination [as observed in Parkinson’s disease (PD), amyotrophic lateral sclerosis, and Huntington’s disease]. There is a bidirectional link between disturbed sleep and dementia. Mazzotti et al. (2014) demonstrated that neurodegeneration was accompanied by sleep disturbances due to a reduction in the amplitude and phase changes of circadian rhythms. Two-third of patients with AD- or PD-related dementia have sleep-related problems, such as frequent daytime napping, nighttime wakefulness, and sundowning (i.e., agitation and disruptive behaviors that occur in the evening or night). Conversely, in terms of duration and quality, lack of sleep could increase neurodegenerative process and aggravate underlying clinical condition. Additionally, poor sleep and sleep disorders, such as insomnia, sleep-disordered breathing, REM sleep behavior disorder (RBD), have been linked to dementia subtypes, such as AD and PD related dementias (Guarnieri et al., 2012; Mazzotti et al., 2014). The difficulty in falling asleep, poor sleep quality, sleep loss, excessive daytime sleepiness, and sleep-disordered breathing can inhibit the inflow of apolipoprotein E (APOE) in cerebrospinal fluid (CSF) and clearance of APOE in interstitial fluid (ISF), which can reduce the removal of Aβ in CSF and increase cerebral Aβ deposition (Branger et al., 2015; Lucey et al., 2017; Carvalho et al., 2018; Lucey et al., 2018). Moreover, Aβ plaques can trigger the mislocalization of aquaporin 4 (AQP4) and decrease CSF influx, thus forming a vicious circle (Peng et al., 2016). Furthermore, apnea or OSA syndrome was related to higher levels of AD-related neuronal injury biomarkers, including P-Tau and T-Tau (Hooghiemstra et al., 2016; Diaz et al., 2017). PD dementia is caused by the aggregation of the protein α-synuclein, deposits of which are known as Lewy bodies (DLBs). Excessive daytime sleepiness, insomnia, and REM sleep behavioral disturbances are some of the most common complaints in PD patients. Moreover, sleep disturbances could increase the level of DLBs, which can aggravate PD development (Barone et al., 2009). Additionally, sleep apnea-associated intermittent brain hypo-oxygenation and inflammation may accelerate the degenerative process in already vulnerable or affected nigral dopaminergic neurons. As such, sleep apnea may exacerbate or accelerate the clinical manifestation of prodromal PD, rather than act as a primary triggering event (Bohnen et al., 2019).

### Effect and Mechanism of Sleep Disturbances on Cardiovascular Disease

Long-term clinical observation shows that the frequency of adverse cardiovascular events is higher in the morning, suggesting that circadian rhythm mechanism plays a significant role in the pathogenesis of cardiovascular diseases. Irregular sleep schedules can lead to milder but chronic circadian disruption, which is widespread in all populations. Specifically, those with irregular sleep patterns may be at higher risk of cardiovascular disease due to disrupted circadian rhythm function (Huang et al., 2020). The physiological mechanisms of disturbed sleep in cardiovascular disease are described in this section. Inflammation is associated with the occurrence and development of cardiovascular diseases (Willeit et al., 2016). A systematic review involving 72 studies concluded that a short sleep time was significantly associated with increased circulating IL-6 levels, and that experimental sleep deprivation was associated with a dramatic increase in the activity of upstream pro-inflammatory molecular pathways (tumor necrosis factor-α mRNA and nuclear factor-κβ activation) (Irwin et al., 2011). Additionally, autonomic nervous system activity, which was characterized by reduced parasympathetic nerve activity and increased sympathetic nerve activity is a risk factor of cardiovascular events (Hillebrand et al., 2013). Similar to the hypothesis that short sleep duration increases the risk of cardiovascular disease through autonomic dysfunction, experimental sleep restriction has been shown to reduce parasympathetic activity and increase sympathetic activity (Tobaldini et al., 2013). Moreover, an early case-control study showed that compared with age- and sex-matched participants without insomnia, patients with insomnia had significantly lower parasympathetic nerve activity (Bonnet et al., 2010). Furthermore, chronic metabolic dysfunctions, such as insulin resistance and impaired glucose tolerance, are main risk factor of cardiovascular disease morbidity and mortality (Huang et al., 2014). A recent study of healthy adults with sleep-deprived lifestyle showed that reduced sleep is a key risk factor for obesity, diabetes, and cardiovascular diseases; moreover, extending sleep duration by approximately 1 h per night improved insulin sensitivity (Killick et al., 2015).

### Effect and Mechanism of Sleep Disturbances on Cognitive Function

Several evidences have shown that sleep disorders could contribute to cognitive decline or cognitive disorders. Studies have shown that various sleep-related parameters, including long sleep latency, poor sleep efficiency or quality, excessive daytime sleepiness, sleep-disordered breathing, delayed sleep phase, and long sleep duration, were associated with cognitive impairments, such as poor attention span, working memory, processing speed, short-term memory, and reasoning, in late life (Lim and Dinges, 2010; Haba-Rubio et al., 2017). Sleep-disordered breathing (SDB), including obstructive sleep apnea (OSA) is associated with 2–6 times higher risk of mild cognitive impairment (MCI) or dementia, and early onset of MCI or dementia (Chang et al., 2013; Leng et al., 2017). Additionally, both insufficient sleep duration (less than 6 h) and excessive sleep duration (more than 9 h) are associated with cognitive impairment, and sleep fragmentation also accelerates the development of preoperative neurocognitive disorder (Ramos et al., 2016). The link between sleep disturbance and cognitive decline and dementia may be related to cortical thinning, a biomarker of several dementia subtypes. A decrease in cortical thickness in the lateral orbitofrontal cortex and inferior frontal gyrus is associated with increased sleep fragmentation, as measured by an Actigraph, and grey matter atrophy in the mPFC is associated with attenuated SOs-spindles coupling and impairment of hippocampal-dependent memory in the elderly. Moreover, short sleep duration may cause hippocampus degeneration through multiple pathways, including changes in neuronal excitability and decreased synaptic plasticity and neurogenesis, which can contribute to cognitive decline (Zimmerman and Aloia, 2006; Macey et al., 2008; Mander et al., 2013; Zarei et al., 2013; Möller et al., 2016). A study showed that sleep deprivation activates neurotoxic complement components C3a and C5a, which affects the hippocampal brain-derived neurotrophic factor (BDNF) pathway and adult neurogenesis, eventually impairing spatial memory (Kessler et al., 2019). Sleep disruption can also directly impair neuronal excitability and synaptic plasticity. Specifically, acute sleep deprivation reduces dendritic spine density in the hippocampal neurons and results in long-term memory impairment, whereas recovery sleep ameliorates spine loss and memory impairment (Havekes et al., 2016).

## Potential Mechanisms of Ketamine in Improving Sleep Quality

Ketamine is a non-competitive antagonist against glutamate NMDARs and has been traditionally used as a dissociative anesthetic. Ketamine contains a mixture of two water-soluble, optical stereoisomers: S (+) and R (−)-ketamine. (Muller et al., 2016). However, whether the antidepressant mechanisms of ketamine is associated with its antagonism of NMDARs is unclear. A previous study showed that although the non-competitive, glutamatergic NMDAR antagonist (R, S)-ketamine exerts rapid and sustained antidepressant effects after a single dose in patients with depression, its use is associated with undesirable side effects. The metabolism of (R, S)-ketamine to (2S, 6S; 2R, 6R)-hydroxynorketamine (HNK) is essential for its antidepressant effects, and the (2R, 6R)-HNK enantiomer exerts behavioral, electroencephalographic, electrophysiological, and cellular antidepressant-related actions in mice. These antidepressant actions are independent of NMDAR inhibition, but involves early and sustained activation of receptors, These same issues may be also apply to sleep effects. (Zanos et al., 2016). The mechanisms through which ketamine improves sleep may be due to its antidepressant effect, its interaction with the circadian system, and its positive neurocognitive effect.

### Ketamine vs. its Enantiomers in Treatment-Resistant Major Depressive Disorder

Ketamine was developed in the early 1960s, and is a popularly abused drug among young individuals and spiritualists because it produces schizophrenia-like symptoms. However, over the years, clinicians and researchers were able to discover its rapid-acting antidepressant effects (Berman et al., 2000; Domino, 2010). Researchers in the United States noticed, in the early 1990s, that patients with chronic depression experienced almost instant relief from depressive symptoms after taking (R,S)-ketamine. Subsequently, several studies replicated the robust antidepressant and anti-suicidal effects of (R,S)-ketamine in treatment-resistant patients with MDD or bipolar depression. Single administration of sub-anesthetic dose of ketamine can induce antidepressant effects in treatment-resistant patients with depression within hours of administration, lasting up to 7 days (Grunebaum et al., 2018; Wilkinson et al., 2018; Yang et al., 2019; Zhang et al., 2019). Ketamine exerts its antidepressant effects by selectively blocking the expression of NMDARs in GABA inhibitory interneurons, resulting in decreased activity of GABAergic interneurons, and de-inhibition of pyramidal neurons, which increases the release of glutamate (an excitatory neurotransmitter) from the synaptic cleft, activates AMPAR, and increases the level of BDNF. These activities result in increased early sleep slow-wave activity during non-REM sleep and improved sleep quality in subjects with treatment-resistant depression. The level of BDNF depends on the regulation of eukaryotic elongation factor 2. Phosphorylated eukaryotic elongation factor 2 can inhibit the translation of BDNF. Ketamine promotes the translation of BDNF by reducing eukaryotic elongation factor 2 kinase activity and inhibiting the phosphorylation of eukaryotic elongation factor 2 (Bjorkholm and Monteggia, 2016; Aleksandrova et al., 2017; Duncan et al., 2017). Moreover, (R, S)-ketamine may significantly increase the release of monoamine transmitters in the central nervous system, promote angiogenesis and synaptic regeneration, and enhance neuronal activity, which may be associated with its antidepressant effects (Ago et al., 2019). Regarding the two enantiomers of (R, S)-ketamine, Zhang et al. first reported that (R)-ketamine had greater potency and longer lasting antidepressant effects than (S)-ketamine in a neonatal dexamethasone-treated model of depression (Zhang et al., 2014). Moreover, (S)-ketamine can cause psychotic reactions, such as depersonalization and hallucinations, in healthy subjects, whereas the same dose of (R)-ketamine did not produce any psychotic symptoms in the same subjects (Wei Y et al., 2021). A PET study showed that (S)-ketamine markedly increased glucose utilization in the frontal cortex and thalamus, whereas (R)-ketamine significantly suppressed glucose metabolic rate in several brain areas, indicating that (S)-ketamine may be responsible for the acute side effects of ketamine (Vollenweider et al., 1997; Zanos et al., 2018). Although adverse effects, such as headache, nausea, and dissociation of (S)-ketamine were observed, intravenous infusion of (S)-ketamine (0.2 and 0.4 mg/kg for 40 min) elicited rapid-acting and sustained antidepressant actions in treatment-resistant patients with MDD (Singh et al., 2016). Another comparative study of (S)-ketamine (0.25 mg/kg by intravenous infusion) and (R, S)-ketamine (0.5 mg/kg by intravenous infusion) demonstrated that (S)-ketamine produced antidepressant effects similar to those of (R, S)-ketamine in treatment-resistant patients with depression, indicating the non-inferiority of (S)-ketamine (0.25 mg/kg) to (R, S)-ketamine (0.5 mg/kg) 24 h after a single infusion (Correia-Melo et al., 2019).

### Regulatory Mechanism of Ketamine on Sleep and Circadian System

Reduced REM, slow-wave sleep and sleep fragmentation are prominent clinical biomarkers of perioperative sleep disturbances and desynchronization of the circadian rhythm (Krenk et al., 2012). For decades, depressive disorders have been related to disrupted sleep patterns and disorganised circadian rhythms. A study of 1,017 participants found that, among 162 functional connections involving sleep-related areas, 39 were also associated with depressive problem scores (Cheng et al., 2018). Ketamine has well-described rapid antidepressant effects in clinical studies of individuals with treatment-resistant MDD, one of the possible antidepressant mechanisms of ketamine may be associated with its actions on clock-gene-related molecules, leading to alterations in the circadian timekeeping of the central clock, and/or with its efects on entrainment circuits that synchronise the central clock with external lighting cycles (Duncan et al., 2017). Ketamine could alter the timing and amplitude of circadian rhythms in rapid responders with increased total sleep, REM sleep, SWA, and slow-wave sleep, potentially (Duncan et al., 2019). Ketamine’s effects on sleep EEG were specific to low frequencies corresponding to the SWA range. Further, ketamine did not increase SWA in waking epochs prior to sleep onset, indicating that the change was sleep-specific. Negligible effects of the ketamine infusion on sleep EEG were measured in bands corresponding to sleep spindles, and alpha or theta frequencies (Duncan et al., 2013). Moreover, BDNF belongs to the neurotrophin family and is produced by astrocytes, in addition to neurons, and the noradrenergic and serotonergic systems play a role in controlling BDNF synthesis (Homberg et al., 2014). Its neurotrophic functions are connected to various physiological functions in the brain particularly relevant in neuroplasticity, memory and sleep (Duman et al., 2014), suggesting its biological role over the entire life span. Duncan et al. also reported that ketamine-induced changes in BDNF levels are associated with its antidepressant efects and increased early sleep SWA during non-REM, as well as with the improvement in sleep quality in subjects with treatment-resistant depression (Duncan et al., 2017).

### Ketamine’s Potential Mechanisms of Action and Potential Predictive Biomarkers Regarding Neurocognitive Function

Apart from its rapid antidepressant effects in patients with treatment-resistant depression, ketamine has been shown to exert neuroprotective effect (either intraoperatively or in the intensive care unit setting with adequate neurocognitive or neuroradiological follow-up), supporting the hypothesis that ketamine may protect cognitive functions. A single dose of ketamine (0.5 mg/Kg) at the induction of anesthesia phase might attenuate postoperative cognitive dysfunction in patients undergoing cardiac surgery and has a potential induction phase that reduces the incidence of postoperative delirium from 31 to 3% (Bell, 2017). Koffler et al. (2007) reported that deep ketamine therapy was effective in relieving chronic pain when patients reached a Ramsay score of 4–5, with ketamine levels of 250–300 µg/dl for at least 4.5 days in a medically-induced coma. Additionally, AD was characterized by glutamatergic hyperactivity receptors, and neuronal and astroglial glutamate transporter dysfunction in Alzheimer’s disease may lead to excess glutamate in the synaptic cleft and excitotoxic neuronal damage. Several investigations have confirmed that ketamine have significant effect on the cerebral activity of animals and humans. The cognitive effect of ketamine may be related to its ability to inhibit cerebral metabolic rate of oxygen, resulting in a decrease in the secretion of excitatory amino acid glutamate neurotransmitter. Therefore, administration of ketamine and other NMDA receptor antagonists, such as memantine, could be used to improve AD symptoms (Masliah et al., 1996; Mallory et al., 1997). Moreover, oxidative stress and protein damage are related to the pathogenesis of AD. Ketamine prevents protein denaturation, lipid peroxidation, and secondary damage of neuronal cells by reducing the formation of free radicals and secretion of various inflammatory mediators, which could improve AD (Lewis et al., 2012; Ye et al., 2013). The hippocampus, which is a significant structure for memory function in humans and spatial memory in rodents (including memory acquisition, consolidation, and retrieval), is mainly affected by AD. A study on the effect of sub-anesthetic dose of ketamine on memory acquisition, consolidation, and retrieval in mice showed that pre-training administration of a sub-anesthetic dose of ketamine inhibited learning and pre-probe administration of ketamine impaired performance. However, post-training administration of ketamine did not affect performance, indicating that sub-anesthetic ketamine can affect memory acquisition and retrieval, but not memory consolidation (Rosenbrock et al., 2011; Moosavi et al., 2012). Moreover, Sun et al. (2011) reported significantly lower level of hippocampal neuronal apoptosis in ketamine-treated rats, which is in accordance with the change in the memory ability and spatial learning in rats. These results confirmed that the ketamine can inhibit hippocampal neuronal apoptosis, and consequently cognitive loss in rat (Sun et al., 2011). However, the neuroprotective mechanism of ketamine in pediatric populations is still poorly understood. Dong and Anand, 2013) reviewed recent data on ketamine neurotoxicity development and proposed strategies for evaluating the safety of ketamine in pediatric patients. Ketamine was found to induce neurodegeneration in the developing brain (Ikonomidou et al., 1999), suggesting the neurotoxicity of ketamine in children. Further studies have indicated that high doses or repeated ketamine doses can induce cell death, especially apoptosis, *in vitro* and in mice, rat, and monkey models (Fredriksson et al., 2004; Amr, 2010). Additionally, ketamine was found to disturb normal neurogenesis of neural stem progenitor cells (NSPCs) in the developing brain (Dong et al., 2012). These findings forced scientists and physicians to reconsider the safety and toxicity of ketamine in pediatric patients. Presently, developmental neurotoxicity is being explored and clarified as a new complication in the clinical use of ketamine in pediatric patients.

## Conclusion and Future Directions

Sleep has recently been recognized as a crucial factor for health and longevity. The biorhythm of daily sleep/wake cycle controls whole-body homeostasis and homeodynamics. Sleep disturbances can cause several physical and psychological disorders, including cardiovascular disease, obesity, depression, and cognitive dysfunction. Thus, improving sleep may reduce the incidence of delirium and cognitive dysfunction. Recently, there has been an increasing research interest on the antidepressant effects of ketamine in patients with treatment-resistant depression and its unique mechanism of action. Particularly, two markers of synaptic plasticity: BDNF levels and EEG sleep slow waves, have been shown to be effective in examining the ability of ketamine to increase synaptic strength. Studies on pharmacology and pharmacokinetics of ketamine and on the predictive biomarkers of ketamine’s effect on sleep and cognitive function have resulted in interesting findings worthy of further research. However, several questions regarding the correlations among sleep, slow waves, and the time course of the emotional response to ketamine are yet to be addressed. For example, sleep slow wave measurements (SWA, amplitude, and slope) seem to be related to a sharp rise in BDNF levels as well as rapid antidepressant response, with a decline 2 days into remission. Conversely, increased total sleep and improved sleep continuity on day 2 are associated with prolonged remission. Further research is needed to identify other signs of plasticity and emotional responses related to the effect of ketamine. If interventions that induce rapid antidepressant effects can increase synaptic strength, then such treatments could have similar effects on EEG sleep slow-wave measurements. Such research would be essential to reveal the key components of the rapid antidepressant response mechanism and to identify novel therapies for treating sleep disturbances.
